# New insights on improved growth and biogas production potential of *Chlorella pyrenoidosa* through intermittent iron oxide nanoparticle supplementation

**DOI:** 10.1038/s41598-020-71141-4

**Published:** 2020-08-24

**Authors:** Mohit Singh Rana, Shashi Bhushan, Sanjeev Kumar Prajapati

**Affiliations:** 1 Enviroment and Biofuel Research Laboratory, Department of Hydro and Renewable Energy (HRED), Indian Institute of Technology Roorkee, Roorkee, Uttarakhand 247667 India; 2 Department of Agricultural and Biosystems Engineering, North Dakota State University, Fargo, ND 58102 USA

**Keywords:** Biofuels, Nanobiotechnology

## Abstract

In the present work, the effect of α-Fe_2_O_3_-nanoparticles (IONPs) supplementation at varying doses (0, 10, 20 and, 30 mg L^−1^) at the intermittent stage (after 12th day of growth period) was studied on the growth and biogas production potential of *Chlorella pyrenoidosa*. Significant enhancements in microalgae growth were observed with all the tested IONPs doses, the highest (2.94 ± 0.01 g L^−1^) being at 20 mg L^−1^. Consequently, the composition of the biomass was also improved. Based on the precedent determinations, theoretical chemical oxygen demand (COD_th_) as well as theoretical and stoichiometric methane potential (TMP, and SMP) were also estimated. The COD_th_, TMP, SMP values indicated IONPs efficacy for improving biogas productivity. Further, the biochemical methane potential (BMP) test was done for IONPs supplemented biomass. The BMP test revealed up to a 25.14% rise in biogas yield (605 mL g^–1^ VS_fed_) with 22.4% enhanced methane content for 30 mg L^−1^ IONPs supplemented biomass over control. Overall, at 30 mg L^−1^ IONPs supplementation, the cumulative enhancements in biomass, biogas, and methane content proffered a net rise of 98.63% in biomethane potential (≈ 2.86 × 10^4^ m^3^ ha^−1^ year^−1^) compared to control. These findings reveal the potential of IONPs in improving microalgal biogas production.

## Introduction

Over the decades, anaerobic digestion (AD) has emerged as one of the established, clean and renewable energy technology for the production of methane-rich biogas^1^. Subsequently, microalgae have been observed as a potential feedstock for biogas production, considering, all the components (viz., carbohydrate, lipid, and protein) are utilized in the process of AD^2^. Ample literatures are available on microalgae-based biogas production that accord a good evidence for its commercial viability^3,4^. Nevertheless, microalgal biogas technology has few limitations. Some of these are owing to the dilute nature of microalgae culture leading to limited availability of biomass. Besides, the poor activity of anaerobic microflora, particularly with microalgal biomass as substrate, reduces the performance of AD^5,6^. Consequently, significant researches have been directed towards improving the microalgae growth to get relatively high biomass concentrations as well as improving the anaerobic digestibility of microalgae through various technological interventions^7^.

In recent years, nanotechnology has come up with the potential to improve bioenergy generation from a range of feedstock, including microalgae. The nanomaterial, including metal nanoparticles (NPs), nanofibers, carbon nanotubes, and other haven been successfully applied in both, microalgal growth and biomass to biofuel conversion^6,8,9^. Among others, iron NPs are observed to be less toxic to microalgae cells^10,11^. In fact, some of the forms of iron NPs (such as α- Fe_2_O_3,_ zero-valent nano iron) are reported to significantly improve the growth of selected microalgae^12^. In particular, iron oxide (α-Fe_2_O_3_) nanoparticles (IONPs) are the least toxic to the microalgae and the environment compared to other forms of iron nanoparticles^10^, and therefore may be a potential material to use at large scale. Further, the iron NPs impart oxidative stress on microalgae that generate reactive oxygen species (ROS) and subsequently affect the biochemical composition of the cell^12,13^. Therefore, the selection of an adequate dose of iron NPs is crucial to improve the microalgal growth and enhance biochemical composition. In a recent report, Kadar et al.^12^ reported 18.75% and 3.57% increase in biomass concentration and lipid accumulation, respectively, for *Isochrisis galbana* by using 100 mg L^−1^ zero-valent nano iron (nZVI). Similarly, Pádrová et al. ^14^ observed an increase of 73.33% in biomass concentration and 58.33% in lipid accumulation for *Desmodesmu subspicatus* by using 5.1 mg L^−1^ nZVI. Likewise, α- Fe_2_O_3_ nanoparticles (IONPs) also showed improvement in the growth of microalgae^15^. However, adding moderate to high doses of iron NPs during the initial growth (particularly during the inoculation stage) may sometimes result in toxicity to the microalgal cells due to very low biomass concentration. For instance, He et al.^15^ documented a reduction in the growth of *Scenedesmus obliquus* at > 5 mg L^−1^ IONPs dose. Such a contrast response is probably due to the excessive stress induced by the nanoparticles on the microalgae cells^16^. Therefore, it is hypothesized to provide IONPs during the late exponential phase, where adequate cells and cellular structure would have developed. Additionally, it can overcome iron-deficient conditions in due growth period and support biomolecule and energy precursors synthesis in further growth phase^13,17^. To the best of authors’ knowledge, no previous attempts have been reported on intermittent IONPs supplementation in microalgae, particularly *Chlorella* spp.

Interestingly, nanoparticles are postulated for effective AD of various feedstock ^18^. High surface to volume ratio of nanoparticles stimulates the metabolic reaction. The electrically (semi) conductive materials can facilitate direct interspecies electron transfer (DIET) between acetogens and methanogens^19^. Therefore, by exciting the microorganisms’ activity, biogas production can be increased^9^. Especially, metal NPs (such as iron, nickel, and cobalt), which can serve as a micronutrient for anaerobic microorganisms, can be valuable^18,20^. Additionally, the iron nanomaterials have been observed in eliminating the inhibitory effects of sulfur and related hazardous compounds in the AD^21,22^. In the past, several attempts were made to assess the effect of nZVI, Fe_3_O_4_ NPs, nickel NPs, cobalt NPs on the AD of various substrates, including sewage sludge^9,18^. Note that the difference among NPs to escalate biogas production lies in its capability to transfer electrons. For example, Fe_3_O_4_/Fe^2+^ and IONPs/Fe^2+^ holds redox potential of − 314 mV, and − 287 mV respectively^19,23^. Nonetheless, in context to IONPs, studies are scant. Ambuchi et al.^24^ reported up to 23% increase in biogas yield by supplying 750 mg L^−1^ IONPs during AD of beet sugar industrial wastewater. Similarly, Juntupally et al.^18^ recorded about 23% enhancement in biogas yield under IONPs supplementation during AD of cattle manure. These findings signify that IONPs can be exploited for enhancement in microalgal biogas yield also. To the best of authors’ knowledge, there are no previous reports available on biogas production from microalgae grown in IONPs supplemented media. Only one report was found on microalgal biomass, where Zaidi et al.^25^ assessed the effect of Fe_3_O_4_ nanoparticles in AD of *Enteromorpha*. Results showed about a 28% increase in biogas yield at 10 mg L^−1^ Fe_3_O_4_ nanoparticles supplementation. Henceforth, IONPs assisted AD was postulated, keeping in mind, the IONPs attached to microalgae cells or leftover in the culture can be directly utilized to enhance the biogas yield.

The present work is the very first attempt to explore the effect of intermittent supplementation of IONPs on *Chlorella pyrenoidosa* growth, composition, and subsequent biogas production. Microalgae biomass concentration, chlorophyll-a concentration, elemental, and biochemical composition was studied to understand the IONPs effect. Empirical formulae were established for the obtained microalgal biomass and theoretical/stoichiometry methane potential was analysed. Further, microalgal biomass, grown in BG11 with intermittent IONPs supplementation, was tested for biogas production following biochemical methane potential (BMP) test protocols.

## Results and discussion

### Microalgae growth at selected IONPs intermittent dose

**Figure 1:**
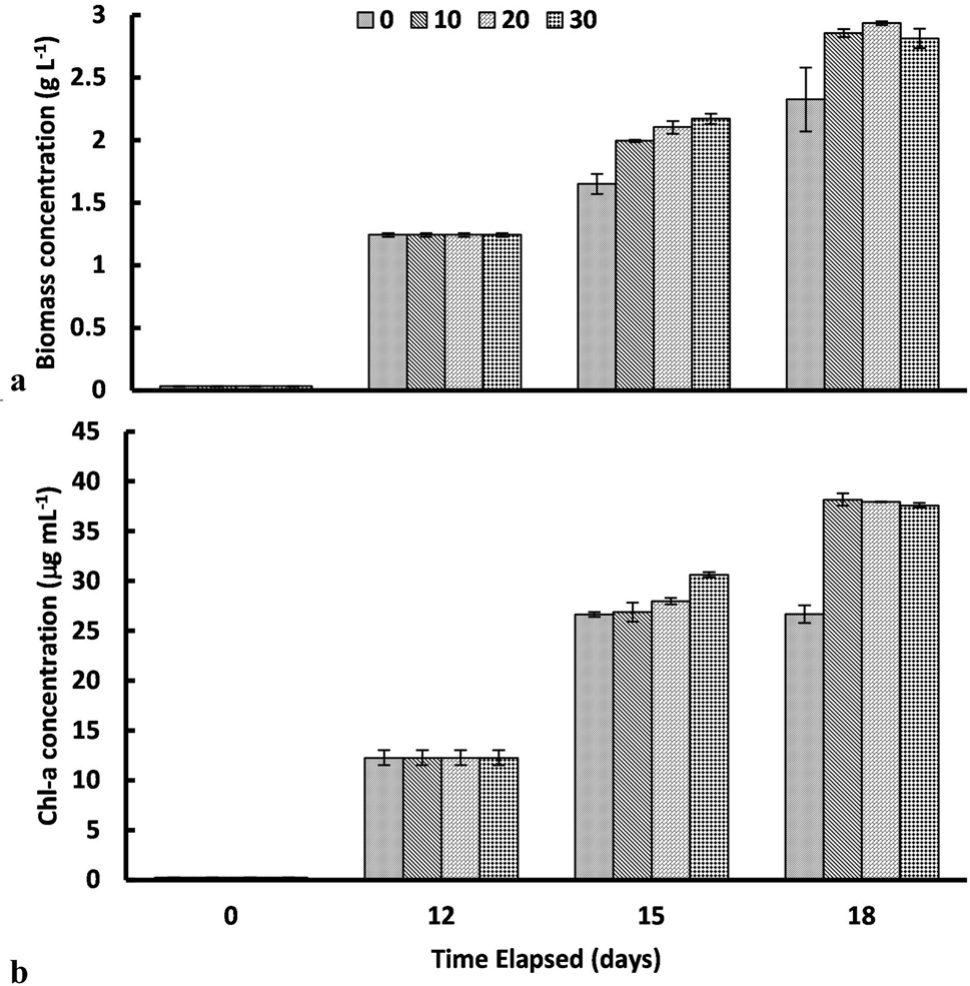
(**a**) Biomass concentration profile and (**b**) chlorophyll-a concentration profile with respect to time for *Chlorella pyrenoidosa* at different initial iron oxide (α-Fe_2_O_3_) nanoparticles (IONPs) doses (data reported as mean with SD in error bars; n = 3).

IONPs effect on *C. pyrenoidosa* growth in terms of biomass and chlorophyll-a (chl-a) concentration in shown in Fig. 1. During the initial growth period (12 days) without IONPs, the observed biomass concentration was 1.24 ± 0.01 g L^–1^. Interestingly, with further growth of algae at different doses of IONPs (13th day onwards), increment in biomass concentration was observed. After 18th day, microalgae concentration showed the highest increase (26.18% over control) using 20 mg L^−1^ IONPs supplementation.

The attained biomass concentration (g L^–1^) was 2.33 ± 0.26, 2.86 ± 0.03, 2.94 ± 0.07 and 2.81 ± 0.07 g L^−1^, respectively, at IONPs dose of 0 (control), 10, 20 and 30 mg L^−1^ (Fig. 1a). A similar pattern was also observed for the chl-a concentration during IONPs supplementation (Fig. 1b). The chl-a concentration after the 12th day of the growth was around 12.26 ± 0.75 µg mL^–1^. The chl-a concentration (µg mL^–1^) significantly increased to 38.17 ± 2.61, 39.94 ± 2.01, and 37.59 ± 2.24, respectively, for IONPs dose of 10, 20 and 30 mg L^–1^ after the 18th day of cultivation.

It is noteworthy that the observed improvement in the growth of microalgae was either higher or at par with the values reported in the literature. For instance, Kadar et al.^12^ achieved up to a 17% increase in the biomass concentration of *Isochrisis galbana* at a dose of 100 mg L^−1^ nZVI. Likewise, He et al.^15^ reported only a 17% increase in the biomass concentration of *Scenedesmus obliquus*. Pádrová et al.^14^ reported 15–73% increase in biomass concentration at a 5.1 mg L^−1^ dose of nZVI for different microalgae species. However, the response of particular microalgal strain differs from each kind of nanoparticle and respective doses. To the best of author’s knowledge, no previous report has focused on IONPs supplementation at an intermittent stage in *Chlorella* cultures. In fact, the studies reporting the synergistic effect of other nanoparticles on *Chlorella* spp. are limited. So far, for *Chlorella* sp. only 8.82% increase in biomass concentration is reported at a dose of 20 mg L^−1^ copper nanoparticles^26^.

On the other hand, there are several reports that showed the toxic effect of the various nanoparticles on *Chlorella* spp. For instance, Ji et al.^27^ examined toxicities of oxide nanoparticles (Al_2_O_3_, SiO_2_, ZnO, and TiO_2_) on the growth of *Chlorella* sp. and, observed highest toxicity with nano-ZnO and nano-TiO_2_. Similarly, silver-nano particles (Ag NPs) also significantly reduced microalgae biomass, and chl-a concentration^28^. Moreover, nickel oxide nanoparticles (NiO-NPs) are reported to cause cellular alterations and hence toxicity to the culture of *Chlorella vulgaris*^29^. Paradoxically, the IONPs tested in the present work resulted in improved growth of *C. pyrenoidosa*. The increase in chl-a and biomass concentration indicates IONPs are being utilized as a micronutrient in the culture resulting in higher photosynthetic activity^30^. The results indicated that all the tested doses of IONPs improve algal growth with 20 mg L^–1^ being significantly higher (p < 0.005). However, to understand the actual role of IONPs in improving microalgae growth, further in-depth experimentations are required. IONPs tracing in cellular pathway and metabolic flux analysis may further reveal the IONPs' fate.

Besides the role of IONPs as a micronutrient, the improvement in the microalgae growth could be attributed to the fact that the IONPs were supplied after the initial growth of microalgae for 12 days. If the IONPs would have been supplied at the start of growth, there were chances of the inhibitory effect on microalgae as the cell concentration was very low in the induction phase, and at this stage, the cells could not combat against the induced toxicity^31^. However, after an initial growth period (12 days), the cell concentration reaches to its moderate value, and cells were probably in an active growth phase. At this stage, the IONPs seem to act as micronutrients rather than showing any toxicity to microalgae. Hence, this innovative strategy of supplementing IONPs after the initial growth of microalgae in nutrient media seems a viable option for higher biomass growth. Overall, IONPs can be utilized as an efficient tool to improve the productivity of *C. pyrenoidosa* during mass scale cultivation for industrial applications, including biofuel production. However, further multifarious research interventions are required to develop the proposed approach for mass scale microalgae cultivation with field trails.

### Microalgae composition and methane potential

**Figure 2:**
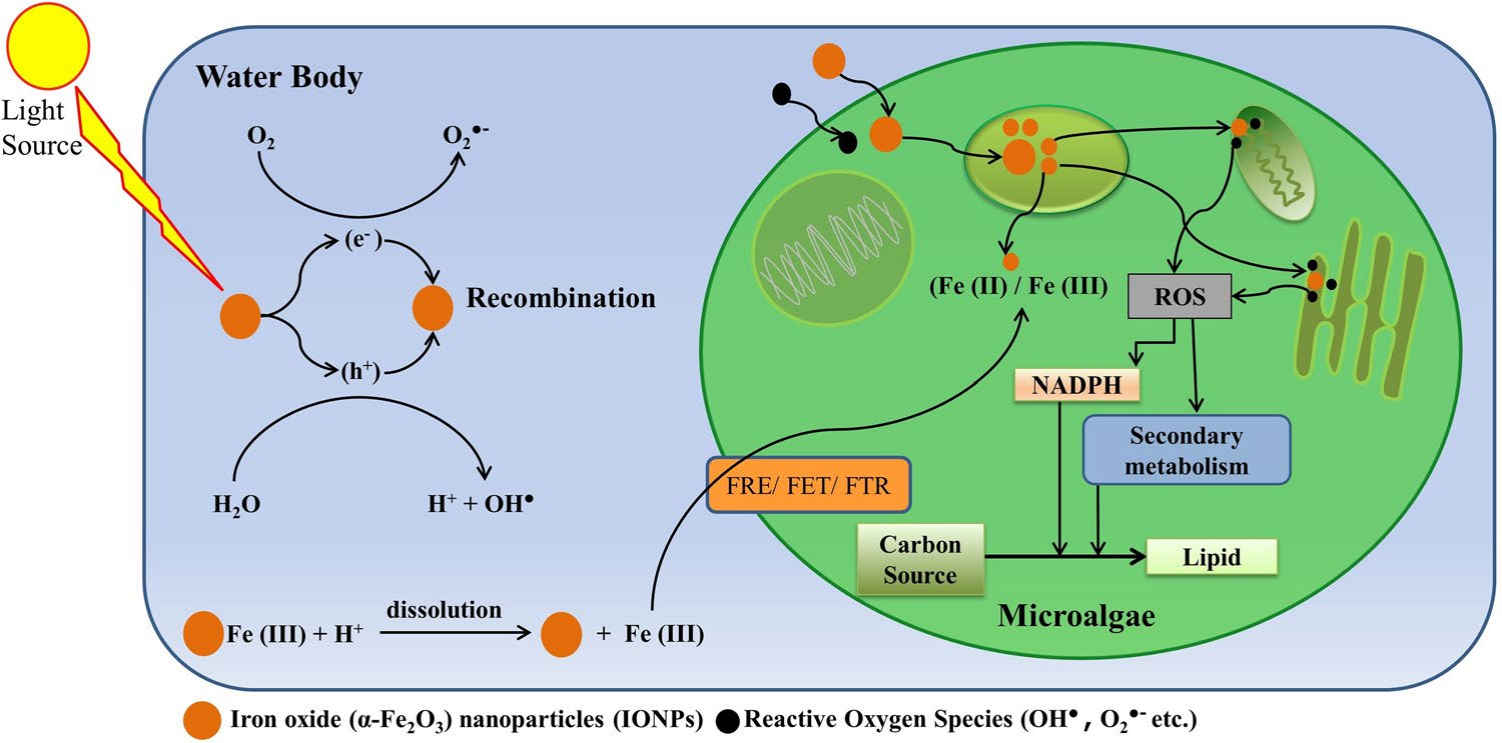
Possible mechanism for nanoparticle assimilation by microalgae (**a**) iron ion transportation through transporter systems present at the cell membrane. (**b**) Direct nanoparticle uptake via passive penetration. FRE/FET/FTR-iron reductases and transporters.

The composition of *C. Pyrenoidosa* grown in BG11 under different IONPs doses are compared in Table 1. The results showed that the IONPs supplementation significantly enhance the VS content. The highest VS (90.23 ± 2.16% of TS) was observed with the microalgae grown at IONPs dose of 20 mg L^−1^. Moreover, the elemental carbon content (C) also increased slightly at 20 and 30 mg IONPs L^−1^ (47.65 ± 1.98, and 47.89 ± 2.01% of TS, respectively) compared to 45% TS for control (Table 1). Whereas, no significant change in the carbon content of algal biomass was observed at IONPs dose of 10 mg L^−1^. The carbohydrate content at each IONPs dose was comparable (23–24% of TS). Interestingly, at an IONPs dose of 20 mg L^−1^, *C. pyrenoidosa* was found with stimulated lipid accumulation (22.74 ± 1.26% of TS) with an increase of 55.22% over control. Whereas, the lipid accumulation (% TS) at IONPs dose of 0 (control), 10 and 30 mg L^−1^, was 14.65 ± 0.60, 19.33 ± 0.29, and 22.06 ± 1.15, respectively. The increased lipid accumulation can be attributed to the cellular stress imposed by the IONPs. Based on the previously published reports^13,32^, a schematic of the lipid stimulation mechanism is depicted in Fig. 2. The improvement in the lipid content during IONPs supplementation was significantly higher or comparable with reported strategies such as nutrient starvation^33^. Overall, the observed biochemical composition of microalgae with IONPs was in line with the reported values suitable for biogas production^34^.

**Table 1:** Biomass composition and estimated methane potential of *Chlorella pyrenoidosa* biomass grown under different IONPs doses.

Parameters	IONP dose (mg L^–1^)			
	0 (control)	10	20	30
Volatile solids (% TS)	82.50 ± 2.23	87.98 ± 3.52	90.23 ± 2.16	88.40 ± 3.68
Ash content (% TS)	17.5 ± 2.16	12.02 ± 3.10	9.77 ± 1.52	11.60 ± 2.92
**Elemental composition (% TS)**				
Carbon	45.90 ± 0.64	45.4 ± 1.52	47.65 ± 1.98	47.89 ± 2.01
Hydrogen	5.95 ± 0.13	7.20 ± 0.63	6.97 ± 0.51	7.11 ± 0.66
Nitrogen	6.88 ± 0.08	6.93 ± 0.33	6.91 ± 0.29	7.21 ± 0.44
Oxygen	23.77 ± 2.57	28.45 ± 1.02	28.70 ± 1.30	26.19 ± 0.95
C/N ratio	6.67 ± 0.08	6.55 ± 0.25	6.89 ± 0.12	6.64 ± 0.26
**Biochemical composition (% TS)**				
Lipid	14.65 ± 0.60	19.33 ± 0.29	22.74 ± 1.26	22.06 ± 1.15
Carbohydrate	24.90 ± 0.69	23.23 ± 0.84	24.12 ± 0.90	23.96 ± 1.20
Protein	41.92 ± 0.63	43.31 ± 2.02	43.19 ± 1.56	45.06 ± 2.26
**Calculated values**				
Empirical formula	C_3.82_H_5.90_ N_0.49_ O_0.85_	C_3.78_H_7.15_ N_0.5_ O_0.82_	C_3.97_H_6.91_ N_0.49_ O_0.65_	C_3.99_H_7.06_ N_0.52_ O_0.54_
COD_th_ (mgO_2_ g^−1^ VS)	2,384.15	2,510.52	2,644.49	2,762.46
TMP (mL g^−1^ VS)	534	573	615	588
SMP (mL g^−1^ VS)	698	738	779	807

Reported as mean ± SD for n ≥ 3

*COD*_*th*_ theoretical chemical oxygen demand, *TMP* theoretical methane potential, *SMP* stoichiometric methane potential.

In line with the elemental composition and biochemical composition, the obtained values for COD_th_, SMP and TMP were observed with notable differences at each tested IONPs dose. The highest SMP value (807 mL g^−1^ VS) was found for microalgae cultivated at IONPs dose of 30 mg L^−1^. However, the highest TMP value (615 mL g^−1^ VS) was observed at IONPs dose of 20 mg L^−1^. Furthermore, the obtained values were significantly higher than that for previously reported for *Chlorella pyrenoidosa* (TMP = 190.41 mL g^−1^ VS)^35^, *Spirulina platensis* (SMP = 278–305 mL g^−1^ VS)^36^, *Scenedesmus* spp. (TMP = 400 mL g^−1^ VS)^37^, and *Chlamydomonas reinhardtii* (TMP = 549 mL g^−1^ VS)^38^. Values for both SMP and TMP at each IONPs concentration advocates enhancement in methane potential. Although SMP estimates the most relevant values as it is based on the empirical formula, unlike TMP, which is based on the fixed chemical formula^34^. However, SMP also estimates values higher than the actual one, since it is based on the elemental composition and cannot differentiate between biodegradable and non-biodegradable compounds^39^. Surprisingly, the obtained TMP and SMP values for IONPs supplemented biomass were relatively higher than that for control. Therefore, the findings clearly revealed that IONPs have a tremendous potential for improving the *C. pyrenoidosa* elemental, biochemical composition, and biomethane potential.

### Biogas production and digestibility of IONPs containing algal biomass

**Figure 3:**
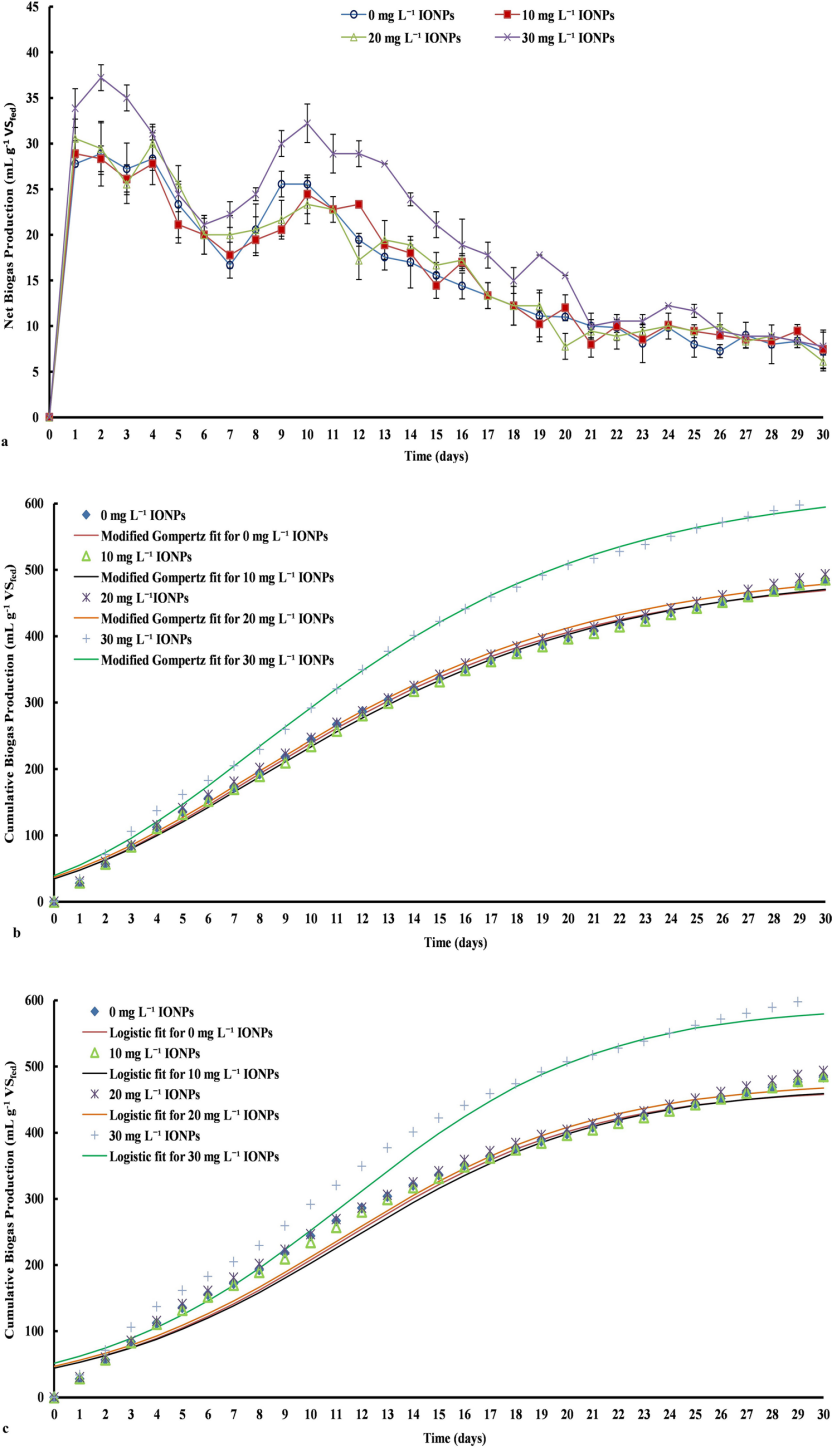
(**a**) Daily net biogas production profile, (**b**) cumulative biogas production profile along with data fitted for modified Gompertz model and (**c**) cumulative biogas production profile along with data fitted for logistic function model with respect to time for iron oxide (α-Fe_2_O_3_) nanoparticles (IONPs) supplemented microalgal biomass (data reported as mean with SD in error bars; n = 3).

Daily net and cumulative biogas production (mL g^–1^ VS_fed_) from biomass of *C. pyrenoidosa* (cultivated with intermittent supplementation of 0–30 mg L^−1^ IONPs) is depicted in Fig. 3. As observed from the daily biogas profiles, for all the tested sets, biogas production started from the first day onwards, without any significant lag (Fig. 3a). The observed biogas production without lag phase could be attributed to the fact that some for microalgal cells might get ruptured during harvesting and preparatory stages of AD experiments^6^. Further, the highest peak (≈ 37.22 mL g^–1^ VS_fed_) in daily biogas profile was observed on 2nd day for the microalgal biomass grown at 30 mg L^−1^ IONPs, followed by peak (≈30 mL g^–1^ VS_fed_) on 4th day for the microalgal biomass grown at 20 mg L^−1^ IONPs. By contrast, weak peaks were observed in the biogas profiles for the microalgal biomass grown at 0 (control) and 10 mg L^−1^ IONPs. For control, in the first 4 days the biogas production increased very slightly from 27.78 to 28.33 mL g^–1^ VS_fed_ and then gradually declined. Similarly, for the microalgal biomass grown at 10 mg L^−1^ IONPs, in the first 4 days, the biogas production was in the range of 28.89–27.78 mL g^–1^ VS_fed_ and subsequently, gradual reduction was observed up to the 7th day. These initial observations indicate the potential role of IONPs at a dose of 30 mg L^−1^ in stimulating the activity of anaerobes.

The biogas production from the microalgal biomass grown at 20 and 30 mg L^−1^ IONPs started increasing again from 6th day onwards and reached a second peak (biogas yield ≈ 32.22 and 23.33 mL g^–1^ VS_fed,_ respectively) on 10th day. Whereas, for the rest of the sets, a slight increase in daily biogas production was observed from 7th to 10th day. Further, the biogas yield decreased gradually (Fig. 3a). The second period of increase in biogas production could be due to the release of microalgal constituents through the hydrolysis of biomass by anaerobic microflora. Moreover, after the highest biogas yield on 2nd day and 10th day for 30 mg L^−1^ IONPs supplemented microalgal biomass, and on the 4th day for 20 mg L^−1^ IONPs supplemented microalgal biomass, sudden fall in biogas production was observed. The sudden fall in biogas yield could be attributed to the volatile fatty acids (VFA) release due to cell disruption which results in the pH drop^40^. Under the acidic pH conditions, the activity of methanogenic bacteria may get declined, and the VFA kept accumulating in the digester^40^. Nonetheless, from the biogas production profile (day 6 to day 10) for 30 mg L^−1^ IONPs supplemented microalgal biomass, it can be clearly seen that a higher dose of IONPs are assisting in improving the biogas yield. A considerably good amount of biogas production continued for all the tested sets (including control) till the 21st day. From 22nd day onwards, the biogas production for all the sets remained in the range of 5–8 mL g^–1^ VS_fed._ Further, for all the tested sets, the pH of the digested biomass obtained after completion of AD was in the neutral range (6.8–7.6). The near to neutral values of pH partially indicated good stability of the AD of microalgal biomass grown in IONPs containing media. However, further detailed investigations on microalgal digestate are warranted to comments on the stability of AD of IONPs containing microalgal biomass in long runs.

Over thirty days of the incubation, the cumulative biogas production was 483.89, 485.56, 493.33, and 605.56 mL g^–1^ VS_fed_ from *C. pyrenoidosa* cultivated with 0, 10, 20 and 30 mg L^−1^ IONPs supplementation (Fig. 3b). The corresponding digestibility for tested sets was calculated to be around 33.00, 33.56, 35.78, and 45.02%, respectively. The observed differences in biogas yield and digestibility of microalgal biomass grown at different IONPs concentration clearly signify the active role of IONPs in improving the digestion of microalgal biomass. As discussed above, the improvement in the biogas production with increasing the IONPs concentration could be attributed to the fact that the iron nanoparticles act as a catalyst for simulating the activity of anaerobic microflora^41,42^. Overall, 25.14% enhancement in biogas production was observed with an increase in IONPs dose to 30 mg L^−1^ as compared to control. The improvement in the biogas production in the present work is comparable with the values reported in the literature on iron nanoparticle supplemented AD of different feedstock, including microalgal biomass (Table 2). For instance, Ambuchi et al.^24^ reported a 22.92% improvement in biogas yield for beet sugar industrial wastewater using 750 mg L^−1^ IONPs. Similarly, Su et al.^21^ reported 5.1–13.2% enhanced biogas yield using 0.10 wt% nZVI in sewage sludge AD. Juntupally et al.^18^ reported up to 23% enhancement in biogas production with IONPs supplementation at a dose of 50 mg L^−1^ during AD of cattle manure. Likewise, in a previous study on sewage sludge, Zaidi et al.^9^ achieved up to 28% increase in biogas yield with Fe_3_O_4_ nanoparticles supplementation at a concentration of 10 mg L^−1^. Paradoxically, Abdelsalam et al.^43^ achieved up to 65.62% enhancement in the biogas yield from raw manure by supplementing 20 mg L^−1^ iron nanoparticles. Further, there is only one paper with a similar effect of iron NPs on AD of microalgal biomass, where Zaidi et al.^25^ documented 28% higher biogas production compared to control (without NPs) from microalgae *Enteromorpha,* by supplementing 10 mg L^−1^ Fe_3_O_4_ nanoparticles in the anaerobic digester. Therefore, there are strong evidences to advocate IONPs' suitability for improving the performance of AD. Further, the present observations, as well as work reported by Zaidi et al.^25^, give clear indications on the potential application of IONPs for improved biogas production from microalgal biomass.

**Table 2:** Comparative study on nanoparticle assisted biogas production.

Nanoparticle type	Size (nm)	Dose (mg L^−1^)	Substrate	Hydraulic retention time (d)	Biogas yield (mL g^–1^ VS_fed_)	Increase in biogas yield (%)	References
IONPs	20	750	Beet sugar industrial wastewater	74	25,144.4	22.92	Ref.^24^
IONPs	< 100	50	Cattle manure	20	160	23	Ref.^18^
Fe_3_O_4_	< 100	10	Sewage sludge	7	624	28	Ref.^9^
IONPs + Fe_3_O_4_	20–40	9	Slaughterhouse waste	28	835.70	37.60	Ref.^46^
Fe_3_O_4_	< 100	10	Microalgae	4.5	289	28	Ref.^25^
IONPs	< 50	30	Microalgae	30	605	25.14	Present work

**Figure 4:**
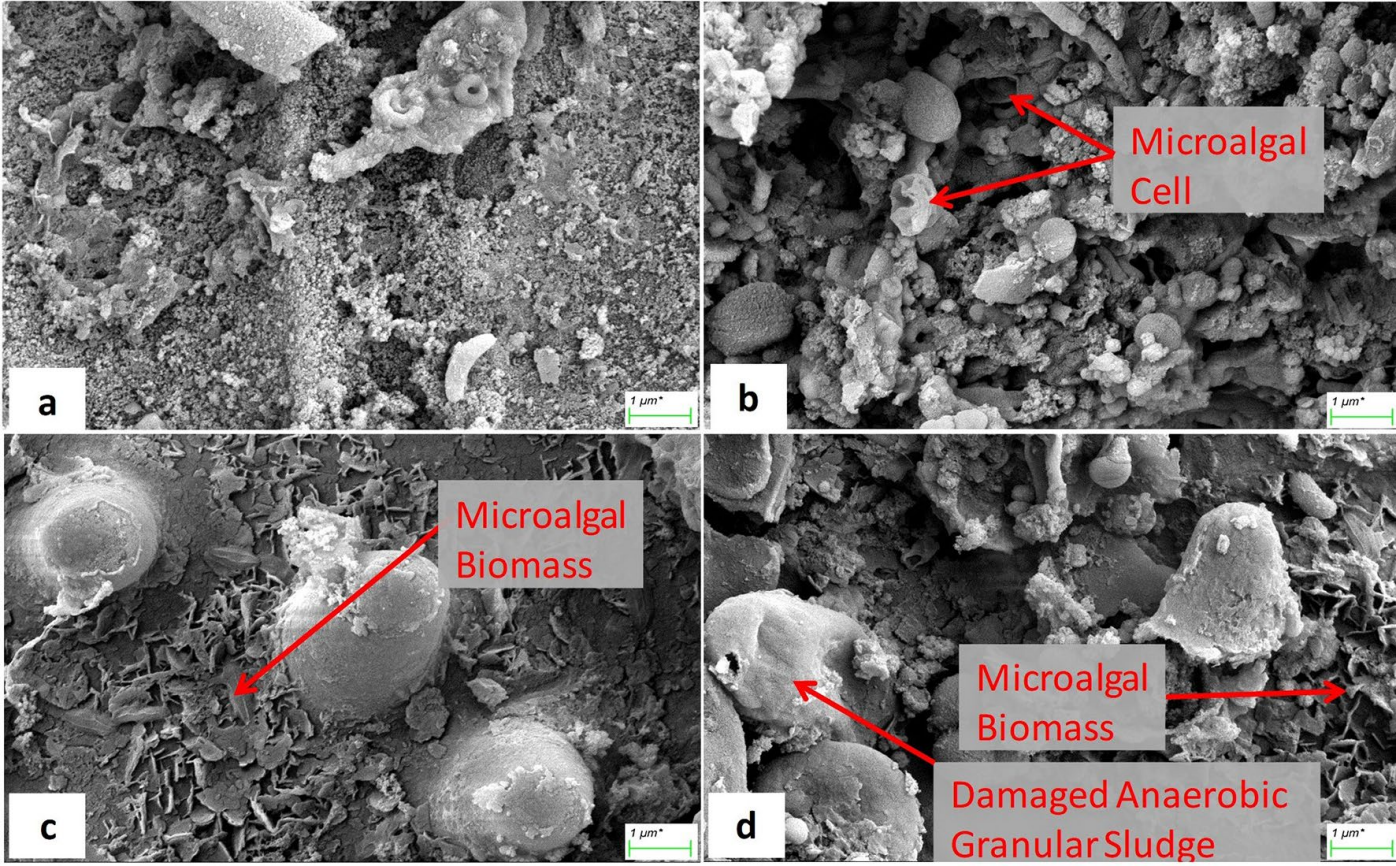
Scanning electron microscopy (SEM) micrographs of (**a**) inoculum and digestate slurry obtained after 30 days of biogas production in different set of iron oxide (α-Fe_2_O_3_) nanoparticles (IONPs) supplemented (varying doses in mg L^−1^) microalgal biomass; (**b**) 0 mg L^−1^, (**c**) 20 mg L^−1^ and (**d**) 30 mg L^−1^ IONPs dose. The SEM micrographs were taken at 25.00 K × magnification.

**Figure 5:**
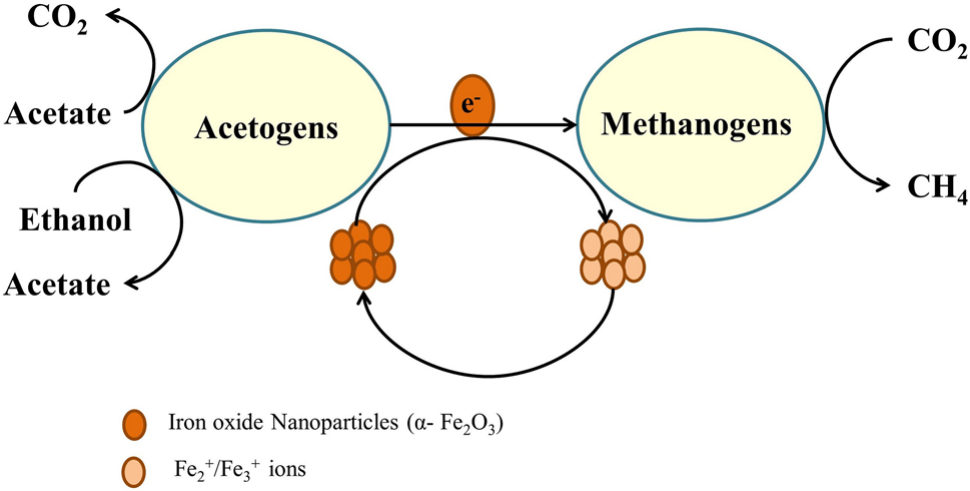
Schematic representation of mechanism for iron oxide (α-Fe_2_O_3_) nanoparticles (IONPs) mediated direct interspecies electron transfer (DIET).

The observed changes in biogas yield were partially supported by SEM micrographs (Fig. 4). At 0 mgL^−1^ dose, squeezed microalgae cells were visible. Moreover, at 30 mg L^−1^ dose, anaerobic granular sludge (AGS) was found slightly defaced. The AGS surface was not as smooth as in the former one. The slight destruction in the sludge granules was perhaps due to the adsorption of nanoparticle on the AGS surface. Because of the high specific area, nanoparticles get adsorbed at the AGS surface due to electrostatic interactions. Subsequently, AGS release some membrane degrading components, such as lactate dehydrogenase and may disintegrate the AGS^20^. At the higher dose of IONPs, shredded biomass was observed (Fig. 4). This partially gives evidence of IONPs utilization by anaerobic microorganisms and efficient assimilation of biomass (or its fermented biomolecules). The iron nanoparticles are postulated to dissolve slowly and supply required iron ions to the anaerobic microorganisms that improve substrate biodegradability rate^44,45^. Moreover, anaerobic microflora utilizes IONPs as conduits for direct electron transfer, specifically from acetogenic to methanogenic microflora. This escalates anaerobic microorganisms’ VFA assimilation rate and therefore improves biogas yield^19^. Similarly, Ambuchi et al.^24^ documented higher biogas and biomethane production rates in the presence of nanoparticles and indicate that the higher biogas yield was because of rapid acetate and propionate utilization as well as low hydrogen accumulation in the reactors. The possible mechanism related to IONPs mediated direct interspecies electron transfer as described by Refs.^19,32^ is depicted in Fig. 5. Further, average methane content in the biogas produced from the microalgal biomass grown with IONPs dose of 0, 10, 20 and 30 mg L^−1^ was 49.04 ± 1.03, 51.02 ± 0.60, 56.50 ± 1.20 and 60.01 ± 1.40% (v/v), respectively. The higher methane content in the biogas coinciding with higher IONPs dose could be due to the partial conversion of CO_2_ to methane through the direct interspecies electron transmission between acetogens and methanogens in the presence of IONPs as catalyst^19,46^.

### AD process evaluation via kinetic models

The fitting of cumulative biogas data to a modified Gompertz model and a logistic function model is shown Fig. 3b, c, respectively. Both of the models showed an improved rate of maximum biogas production (R_m_) at elevated IONPs doses in line with the experimental biogas yield. The modified Gompertz model showed R_m_ values of 23.09, 23.65, 23.72 and 29.77 mL day^−1^ for 0, 10, 20 and 30 mg L^−1^ IONPs supplemented microalgal biomass, respectively. Likewise, R_m_ values for 0, 10, 20 and 30 mg L^−1^ IONPs supplemented microalgal biomass were 23.31, 23.72, 23.76 and 30.27 mL day^−1^, respectively, via the logistic function model. From the kinetic study it can be clearly seen that IONPs supplementation reduced the lag phase time and improved the biogas production rate (Table 3). The modified Gompertz model was best fitted with the experimental data (R^2^ = 0.995–0.997), whereas the logistic function model showed 0.987–0.991 R^2^ value for all the experimental conditions. The modified Gompertz model was observed to be more suitable in this case over logistic function model. Henceforth, based on the modified Gompertz model, for the microalgal biomass grown in 30 mg L^−1^ IONPs supplemented media, up to 630.80 mL biogas g^–1^ VS_fed_ can be anticipated over a period of 30 days with a slight lag phase (λ) of 0.5426 d. The biogas production values obtained for the present study were significantly higher compared to the previously reported values. For instance, Zaidi et al.^47^ had reported 374.528 mL maximum biogas potential, with an R_m_ value of 3.733 mL h^−1^ and lag phase (λ) of 0.672 h, while using 10 mg L^−1^ Fe_3_O_4_ nanoparticles’ concentration in microalgal biomass during AD. Hence, it is clear from the study that elevated levels of iron nanoparticles in microalgal biomass AD has potential to enhance the biogas productivity enormously.

**Table 3:** Parameters of the kinetic models.

Parameters	Modified Gompertz model				Logistic function model			
	IONP dose (mg L^–1^)							
	0 (control)	10	20	30	0 (control)	10	20	30
B_P_ (mL g^–1^ VS_fed_)	495.20	501	507.10	630.80	467.90	470.70	478.50	529.9
R_m_ (mL day^−1^)	23.09	23.65	23.72	29.77	23.31	23.72	23.76	30.27
λ (d)	0.8643	0.8641	0.7033	0.5426	1.277	1.33	1.103	1.003
R^2^	0.996	0.996	0.995	0.997	0.988	0.988	0.987	0.991

*B*_*p*_ maximum biogas production potential, *R*_*m*_ rate of maximum biogas production, *λ* lag phase, *R*^*2*^ coefficient of determination.

### Energy analysis and future application potential

Based on reported techno-economic feasibility for microalgae cultivation at mass scale, multiple raceway pond of 25 cm depth were considered, installed in an area of 1 hectare^48^. Due to variation in the environmental conditions, net microalgae biomass productivity in a raceway pond was assumed to be 62.5% of the closed photobioreactor system^49^. Hence, for the raceway pond of given depth, 93.01 tons ha^−1^ year^−1^ net biomass productivity was estimated for the microalgal biomass containing 20 mg L^−1^ IONPs dose, followed by 90.50, 89.16, and 73.70 tons ha^−1^ year^−1^ at IONPs dose of 10, 30, and 0 mg L^−1^ (control), respectively. Moreover, based on the BMP test, the highest biogas productivity was estimated to be 4.77 × 10^4^ m^3^ ha^−1^ year^−1^ for 30 mg L^−1^ IONPs supplemented microalgal biomass (Table 4). Based on the present observations, it can be concluded that the IONPs enhanced the biogas productivity along with improvement in methane content. Therefore, cumulative enhancement in microalgal biomass, biogas, and methane content for microalgal biomass grown at 30 mg L^−1^ IONPs supplementation proffered a net rise of 98.63% in biomethane production as compared to control. Consequently, the estimated biomethane potential was found to be 2.86 × 10^4^ m^3^ ha^−1^ year^−1^. Similarly, for the predicted values from the modified Gompertz model, up to 2.98 × 10^4^ m^3^ ha^−1^ year^−1^ biomethane production can be achieved using 30 mg L^−1^ IONPs supplemented microalgal biomass. The estimated values were significantly higher than the previously reported value (12,128 m^3^ ha^−1^ year^−1^) for mixed microalgae culture cultivated in carpet industry wastewater^50^. It is noteworthy that the use of IONPs in an adequate amount in microalgae cultivation and sequentially, in biogas production can enhance the net yield in a confounded way. Furthermore, by using an adequate amount of IONPs, up to 1,025.19 GJ of renewable energy ha^−1^ year^−1^ (equivalent to 0.29 GWh ha^−1^ year^−1^) can be generated using *C. pyrenoidosa* (Table 4). Henceforth, *C. pyrenoidosa-*based biogas production holds potential to meet power demand from renewable resources. Further, along with IONPs supplementation, large scale feasibility can be assessed using wastewater as growth media for *C. pyrenoidosa* and CO_2_ as a carbon source for further enhancement in biomass productivity^51^. In fact, flue gas having 6–12% CO_2_ content can be used^48^, and further green economy can be built through carbon trading with flue gas producing industries, such as thermal power stations. Along with wastewater, liquid digestate obtained after AD can be used as a nutrient source for microalgae cultivation^52^. Further, the preliminary inductive coupled plasma (ICP) based analyses showed that the liquid digestate obtained from IONPs supplemented microalgal biomass contains around 25–35% leftover IONPs (or other species of iron nanoparticles). The recycling of IONPs companion liquid digestate back to microalgae cultivation would be beneficial to cut down the overall cost and reliance on extraneous commercial nutrient resources^53^. Additionally, it is likely that the IONPs aggregate and remain with solid digestate under static conditions^54^. However, further analyses including SEM–EDX, ICP-MS, TEM and X-ray absorption near-edge spectroscopy of the solid as well as liquid digestate are warranted for confirming both the exact amount and dominating forms of leftover iron nanoparticles. The solid digestate having leftover IONPs (or iron) may serve as an efficient bio-fertilizer. Indeed, previous studies perceived that iron nanoparticle companion fertilizer would improve the bioavailability of iron to the plant^55^. In turn, IONPs companion fertilizer would be beneficial to overcome the iron deficiency and would assist in increasing plant yield^56^. Furthermore, the nanoparticles can assist in nitrogen and phosphorus fixation in plants^57^. Nevertheless, multifarious scientific interventions are required to account IONPs availability as well as any changes in physical or chemical properties and reusability performance. In this way, IONPs application on microalgae can be a boon for energy, agriculture, environment, and economy.

**Table 4:** Estimated bioenergy potential for IONPs supplemented *Chlorella pyrenoidosa* cultivated in hypothesized multiple open raceway ponds (dimension: L × W × D = 219 × 20 × 0.25 m, each).

Parameters	Experimental bioenergy potential					Predicted bioenergy potential			
	IONP dose (mg L^–1^)								
	0 (control)	10	20	30	0 (control)		10	20	30
Biomass productivity (tons h^−1^ year^−1^)	73.70	90.50	93.01	89.16	73.70		90.50	93.01	89.16
Volatile solids (% of total solids)	82.50	87.98	90.23	88.40	82.50		87.98	90.23	88.40
Biomass productivity (VS tons ha^−1^ year^−1^)	60.80	79.63	83.92	78.82	60.80		79.63	83.92	78.82
Biogas yield (m^3^ kg^−1^ VS_fed_)	0.48	0.49	0.49	0.61	0.50		0.50	0.51	0.63
Net biogas yield (10^4^ m^3^ ha^−1^ year^−1^)	2.94	3.87	4.14	4.77	3.01		3.99	4.26	4.97
Methane content (%)	49	51	56	60	49		51	56	60
Net biomethane production (10^4^ m^3^ ha^−1^ year^−1^)	1.44	1.97	2.31	2.86	1.47		2.03	2.38	2.98
Net energy yield^a^ (GJ ha^−1^ year^−1^)	516.12	705.90	829.99	1,025.19	528.19		728.35	853.15	1,067.93

The annual data was estimated based on the experimental values and predicted values obtained from modified Gompertz model.

^**a**^Considering energy value for biomethane^68^ to be 35.80 MJ m^−3^.

## Materials and methods

### Algal culture and reagents

Microalgae *Chlorella pyrenoidosa* (NCIM 2,738) was procured from National Centre for Industrial Microorganism, Pune, India. The microalgae culture was maintained in BG 11 broth (HiMedia, Cat. No. M1541) under LED light (intensity ≈ 4,000 lx, and 18:6 h light/dark cycle), at room temperature (25 ± 2 °C). The iron oxide nanoparticle (α-Fe_2_O_3_-NPs) powder, (average particle size < 50 nm), termed as IONPs, was procured from Sigma-Aldrich (Cat. No. 544884).

### Experimental procedure

Initially, the freshly grown *C. pyrenoidosa* (OD_680_ ≈ 2.0) was inoculated (10% v/v) into 50 mL of sterile BG-11 broth. The flasks were then incubated for 12 days under controlled conditions as described above. On the 13th day, the grown microalgae culture was supplemented with varying concentrations of IONPs (0, 10, 20, and 30 mg L^–1^) in separate flasks and allowed to grow further for the next 6 days. Microalgae growth was measured in terms of biomass concentration and chlorophyllaa (chl-a) concentration for 0th day, 13th day (before IONPs supplementation), and then at an interval of 3 days for rest of period. After the 18th day, the microalgal biomass was harvested by centrifugation at 6,900 rpm (7,350 g) for 10 min. The harvested biomass from different sets were dried and process for elemental (CHN) and biochemical (lipid, protein, and carbohydrate) composition analyses. Based on the composition, theoretical chemical oxygen demand (COD_th_), theoretical methane potential (TMP), and stoichiometric methane potential (SMP) were estimated for the algae grown under different IONPs supplementation following methodology reported in previous work^58^.

For AD studies, *C. pyrenoidosa* was cultivated in a fabricated cylindrical polyacrylic photobioreactors (dimension: length = 60 cm, diameter = 20 cm) with a working volume of 20 L. The PBR was illuminated under LED light (≈ 4,000–4,500 lx), with 18:6 h light/dark cycle. Culture mixing was achieved through bubbling using an air pump at a flow rate of 1.5 L min^–1^. The microalgae cultivation was carried out at a temperature of 25 ± 2 °C. Similar to shake flask studies, after 12 days of microalgae growth, each PBR was supplemented with IONPs at a dose of 0, 10, 20, and 30 mg L^–1^, separately. The microalgae were then allowed to grow for the next 6 days with added IONPs. After completion of growth, microalgae biomass was harvested as a concentrated slurry using gravity settling in a fabricated Imhoff tank by keeping the culture static for overnight. Along with microalgae, all the IONPs were tend to settle down due to higher density than microalgae and IONPs tendency to form aggregates under static condition^54^. The concentrated microalgal slurry obtained after decanting the supernatant was subsequently used in AD studies following the biochemical methane potential (BMP) test^59^. The inoculum was prepared using digestate slurry, collected from running cattle dung based biogas plant. The inoculum was first acclimatized with microalgae biomass in batch anaerobic reactor at 37 ± 1 °C, for 30 days. The specific methanogenic activity (SMA) of the acclimatized inoculum was 92.16 L CH_4_ kg^−1^ VSS day^−1^. The BMP tests were performed in 250 mL amber colour bottles (working volume 180 mL), equipped with hermetically sealed stoppers. Substrate to inoculum ratio was kept 1:3, with a substrate load of 5 g VSL^–1^^58^. BOD bottles having inoculum only were used as negative control. The BOD bottles were incubated at 37 ± 1 °C in an incubator, and biogas production was measured after every 24 h for 30 days.

### Analytical methods

Microalgae growth was estimated by recording the absorbance of 1 mL microalgae culture at 680 nm using a spectrophotometer (UV–VIS, Lamda 365, Perkin Elmer). Further, the absolute value biomass concentration (g L^−1^) was calculated using the standard calibration curve61 (Eq. 1) prepared between microalgal dry cell weight and culture absorbance at 680 nm.

1 $$ B = 0.4469 \times A_{680} + 0.003 $$

where, A_680_ and B is the absorbance at 680 nm and biomass concentration (g L^–1^), respectively.

Further, 1 ml of microalgae culture was withdrawn from the culture broth for (chl-a) estimation, and centrifuged at 6,900 rpm (7,350*g*) for 10 min at room temperature. Further, it was washed thrice using deionized water through repeated cycles of centrifugation and resuspension. The washed biomass pellets were resuspended in 1 mL methanol. The centrifuge tubes having methanol suspended algal cell pellets were tightly sealed using parafilm and heated at 60 °C in a water bath for 30 min. The sample was then cooled to room temperature, and the absorbance was recorded at 652, 665.2, and 750 nm using a spectrophotometer (Perkin Elmer, UV–Vis, Lamda 365). Porra’s equation^61^ was used to calculate the final chl-a concentration in μg mL^−1^.

The total solids (TS) in microalgae and digestate slurry (inoculum) was estimated by drying each sample separately at 70 °C in a hot air oven till constant weight. The Volatile solid (VS) content was estimated through burning the dried biomass samples in a muffle furnace at 550 °C for 2 h^62^. The elemental (C, H, N) composition of the microalgal biomass was determined using CHNS/O analyzer (2400, Perkin Elmer Series II). The lipid content in the biomass was determined using modified Bligh, and Dryer’s method^63^ Carbohydrate content was estimated by the phenol–sulfuric acid method given by Dubois et al.^64^. The protein content of the biomass was calculated using the biomass nitrogen content, and the nitrogen to protein conversion factor of 6.25 ^65^. The SMP and TMP were estimated using the Eqs. 2 and 3, adopted from Sialve et al.^34^.

2 $$ SMP = \frac{1}{8}\left( {\frac{4a + b - 2c - 3d}{{12a + b + 16c + 14d}}} \right) V_{m} $$

where, V_m_ is the molar volume of methane at STP.

3 $$ TMP = \frac{1}{100}\left( {A \times C_{L} + B \times C_{P} + C \times C_{C} } \right) $$

where, A, B, C are the specific methane yield of lipid, protein, and protein, respectively and C_L_, C_P_, C_C_ are the lipid, protein, and carbohydrate composition (% on TS basis) of the microalgal biomass.

During the BMP test, biogas production from the experimental bottles was estimated at a regular interval of 24 h using acidic water pH < 1.5) displacement method^59^. The actual biogas production from the experimental sets was calculated using the following equation:

4 $$ B_{actual} = B_{\exp erimental} - B_{control} $$

where, B_actual_, B_experimental_ and B_control_ are actual biogas volume (mL), biogas obtained from each experimental sets and biogas obtained from control.

The methane content in the biogas was determined using gas chromatography, equipped with thermal conductivity detector^58^. After the BMP test, the solid digestate at each IONPs dose was processed for morphological analysis using scanning electron microscope (SEM). For sample preparation, the digestate was lyophilized, mounted on aluminium stubs and coated with gold using plasma spraying. Further, the prepared sample was analysed using a SEM (GeminiSEM 500, Zeises).

### Kinetic models for biogas production

The performance of AD was evaluated mathematically via fitting the cumulative biogas data in modified Gompertz model^66^ (Eq. 5) and logistic function model^67^ (Eq. 6). GraphPad Prism software; version 8 (https://www.graphpad.com/scientific-software/prism/) was used for data computation and all kinetic parameters were determined for the both models.

5 $$ B = B_{P} . exp \left( { - exp \left( {R_{m} . \frac{2.7183}{{B_{P} }} . \left( {\lambda - t} \right) + 1} \right)} \right) $$

6 $$ B = \frac{{B_{P} }}{{1 + exp \left( {4 . R_{m} . \frac{\lambda - t}{{B_{p} }} + 2} \right)}} $$

where, B, B_P_, R_m_, λ and t are cumulative biogas volume (mL), ultimate biogas production potential (ml), rate of maximum biogas production (mL day^−1^), lag phase (d) and digestion time (days), respectively.

### Statistical analysis

All the experiments were carried out in triplicates unless stated otherwise. Data was analysed using the statistical software, GraphPad Prism software; version 8 (https://www.graphpad.com/scientific-software/prism/). The P-value for all tests was ≤ 0.05. The values were reported as either mean ± SD in the text or mean with error bar in the graphs.

## Conclusion

The present study revealed the potential of IONPs supplementation at the intermittent stage for enhanced *Chlorella pyrenoidosa* growth and biogas production potential. IONPs at a dose of 20 mg L^−1^ was adequate to improve biomass productivity. Further, a 30 mg L^−1^ dose of IONPs yielded highest biogas production with improved activity of anaerobes. The sequential use of IONPs in an adequate amount is recommended for microalgae cultivation and biogas production. The experimental results were in line with the elemental composition, biochemical composition, COD_th_, SMP, and TMP. Further, a detailed feasibility analysis may open a wide window for methane-rich biogas generation.
